# Supplementary material for: Association of orthostatic blood pressure response with incident heart failure: The Framingham Heart Study
Source: PLoS One. 2022 Apr 22;17(4):e0267057. doi: 10.1371/journal.pone.0267057 (PMC9032405; doi:10.1371/journal.pone.0267057)
Supplement: S1 Table — (DOCX) [file pone.0267057.s001.docx]

**Supplementary Material**

**S1 Table:** Characteristics of study sample by Orthostatic Blood Pressure Response Category

| **Characteristics** | **OH**  **(n=274; 14%)** | **OHT**  **(n=399; 21%)** | **Neither (n=1,241;65%)** |
| --- | --- | --- | --- |
| Age, years | 74±7 | 71±6 | 71±7 |
| Female sex | 181 (66%) | 229 (57%) | 749 (60%) |
| Body Mass Index, kg/m^2^ | 26.2±4.6 | 26.5±4.3 | 26.5±4.3 |
| Seated Systolic Blood Pressure, mm Hg | 145±22 | 141±19 | 142±19 |
| Seated Diastolic Blood Pressure, mm Hg | 77±10 | 77±10 | 78±10 |
| Heart rate, min^-1^ | 70.4±13.6 | 70.9±13 | 71.3±12.3 |
| Hypertension treatment | 148 (54%) | 172 (43%) | 522 (42%) |
| TC/HDL-C, mg/dL | 4.9±1.6 | 4.9±1.6 | 5.0±1.7 |
| Lipid-lowering treatment | 5 (2%) | 7 (2%) | 16 (1%) |
| Diabetes Mellitus | 21 (8%) | 22 (6%) | 77 (7%) |
| Glucose-lowering treatment | 18 (7%) | 20 (5%) | 69 (6%) |
| Current Smoker | 50 (18%) | 81 (20%) | 212 (17%) |

Data are given as mean ± SD or n (%). OH indicates orthostatic hypotension; OHT, orthostatic hypertension; TC/HCL, total cholesterol/high-density lipoprotein cholesterol.
