# Supplementary material for: Association of orthostatic blood pressure response with incident heart failure: The Framingham Heart Study
Source: PLoS One. 2022 Apr 22;17(4):e0267057. doi: 10.1371/journal.pone.0267057 (PMC9032405; doi:10.1371/journal.pone.0267057)
Supplement: Attachment — Submitted filename: PLOS ONE Response to Reviewers_March 14 2022.docx [file pone.0267057.s002.docx]

**PONE-D-22-03355**

**Association of orthostatic blood pressure response with incident heart failure: The Framingham Heart Study**

**Review Comments to the Author**

**Reviewer #1:** **The study evaluates the effect of orthostatic hypotension and hypertension with heart failure risk in the community and the relations with HF subtypes [HF with reduced ejection fraction and HF with preserved ejection fraction]. Data derived from 1,914 participants enrolled in the 17 cycle between 1981–1984 of the Framingham Heart Study Original Cohort followed until December 31, 2017 for incident HF or death. OH or OHT, were defined as a decrease or increase, respectively, of 20/10 mmHg in systolic/diastolic BP upon standing from supine position. At baseline, 1,241 participants had a normal BP response, 274 had OH, and 399 had OHT. On follow-up (median 13 years) we observed 492 HF events (292 in women; 134 HFrEF, 116 HFpEF, 242 HF indeterminate EF). Compared to the referent, participants with OH [n= 84/274 (31%) HF events] had a higher HF risk (Hazards Ratio [HR] 1.47, 95% CI 1.13—1.91). Moreover, OH was associated with a higher HFrEF risk (HR 2.21, 95% CI 1.34—3.67). OHT was not associated with HF risk. The topic is important and the manuscript is relevant even if is confirmative of a known association. The study methodology is eccelent. Of interest is the association of OH with HFrEF. Data supports conclusions, the manuscript is well written and I have no suggestions to improve it.**

**Response:** We thank the Reviewer for the thoughtful comments.

**Reviewer #2:** **Orthostatic hypotension (OH) and hypertension (OHT) are aberrant blood pressure regulation conditions, expression of dysregulation of heart rate, cardiac output or peripheral vascular resistance, that are associated with higher cardiovascular disease risk. Authors evaluated the role of OH and OHT in HF risk founding that OH was associated with a higher risk of developing HF and HFrEF, but not HFpEF. In contrast, OHT was not associated with HF risk.
The paper has merit, but there are points that deserve a major revision.**

**Comment #1. In “Outcomes of Interest” section, no information is given about definition of incident HF subtypes. Please describe how the presence of a reduced LV systolic function was determined by echocardiography.**

**Response:** We thank the Reviewer for this comment. The presence of a reduced LV systolic function was determined by reviewing the hospitalization and echocardiography reports we obtain from the participants’ physicians when they are hospitalized with a HF event. We have now revised the text as follows:

Page 8: “The presence of a reduced LV systolic function was determined by reviewing the hospitalization and echocardiography reports we obtain from the participants’ physicians when they are hospitalized with a HF event.”

**Comment #2. The data on ejection fractions of 242 patients with HF are missing. Considering that these represent 49% of the population with HF emphasize this important limitation of the study**.

**Response:** We thank the Reviewer for raising this important point. Examination cycle 17 took place during the years 1981-1984, where echocardiography was not used as frequently for HF events; hence the large number of participants with missing information on ejection fraction. We have now added the following in the Strengths and Limitations section in the revised version of the manuscript:

Page 14:” Finally, echocardiography was not used as frequently during the period when examination cycle 17 took place, explaining the large number of participants with missing information for ejection fraction at the time of HF. ”

**Comment #3. As reported by the authors, the association between OH and HF has already been described, but in contrast to prior studies in which the association of OH and risk of HF was lost or attenuated after adjustment for traditional risk factors, investigators observed in this work higher incidence of HF and sustained statistical significance after adjusting for all covariates (age, sex, BMI, seated SBP, seated DBP, antihypertensive treatment, smoking, diabetes, etc). Please describe the clinical and demographic differences in the sample in relation to the BP response to posture change (OH, OHT, neither OH nor OHT).**

**Response:** We thank the Reviewer for this comment, we present the participants characteristics by OH/OHT status in Supplementary Table 1 (S1 Table).

**Comment #4. As reported, the elderly sample, with a mean age of 72 years, may have contributed to higher prevalence of OH and higher incidence of HF. What about frailty status? Please see and discuss “Liguori I. et al. Orthostatic Hypotension in the Elderly: A Marker of Clinical Frailty? J Am Med Dir Assoc. 2018 Sep;19(9):779-78”.**

**Response:** We thank the Reviewer for this very important observation. However, we do not have data available for frailty at the examination cycle 17. We acknowledge the importance of frailty by adding the following text in the revised version of the manuscript and also citing the reference by Liguori et al. that the Reviewer kindly suggests:

Page 13: “We should note that OH could be a marker of frailty, as discussed by Liguori et al; however, we do not have frailty data available at examination cycle 17 to evaluate this hypothesis.”

**Comment #5. Authors suggest that OH may serve as a non-traditional factor associated with higher HF and HFrEF risk, even among patient with hypertension. Please discuss if the effect of OH on HF could depend on a non-optimal use of anti-hypertensive therapy.**

**Response:** We thank the Reviewer for this important comment. Unfortunately, we do not have available data on compliance regarding use of medications so we are not able to evaluate this premise. We added the following text in the Discussion section of the revised version of the manuscript:

Page 13: ”It remains unknown if the role of OH on HF risk is modified by optimal compliance with antihypertensive therapy or medications. Data on medication compliance are not available for the present investigation; it is important to evaluate the effect of medication compliance on the observed association in future studies.”
